# Immunogenicity of a monovalent 2009 influenza A (H1N1) vaccine in infants: randomized, observer-masked, single-center clinical study

**DOI:** 10.1186/2193-1801-3-397

**Published:** 2014-07-31

**Authors:** Shilei Wang, Jinrong Dong, Wenqing Chai, Fangjun Li, Shuqiao Wang, Bing Sun, Ze Chen

**Affiliations:** Shanghai Institute of Biological Products, Shanghai, 200052 China; Hunan Provincial Center for Disease Prevention and Control, Changsha, Hunan, China; Institute Pasteur of Shanghai, Shanghai Institutes for Biological Sciences, Chinese Academy of Sciences, Shanghai, China

**Keywords:** Infant, Influenza, Vaccine, Immunogenicity

## Abstract

**Abstract:**

The aim of this study is to further investigate the immune response of the inactivated split-virion vaccine for infants. We tested the immunogenicity and safety of the inactivated split-virion vaccine in infants, aged 6–35 months, for a randomized, observer-masked, age-stratified clinical study. We randomly divided subjects into three groups: 7.5 μg, 15 μg of hemagglutinin antigen dosage groups and seasonal influenza vaccine for children dosage group in a 2 dose regimen. A serologic analysis was performed at baseline and on day 21 and 42. 312 infants received a single dose injection of vaccine and 265 (84.94%) infants received two doses injection of vaccine. Adverse reactions were mostly mild or moderate. Among the subjects who received 7.5 μg and 15 μg of vaccine for a single dose injection, the rate of hemagglutinin inhibition titer of 1:40 or greater were 52.48% (95% confidence interval (CI) 42.83 ~ 61.95) and 61.11% (95% CI 50.78 ~ 70.53), respectively. Among the subjects receiving 7.5 μg and 15 μg of vaccine for two doses injection, the rate of hemagglutinin inhibition (HI) titer of 1:40 or greater were 90.10% (95% CI 82.73 ~ 94.53) and 94.44% (95% CI 87.64 ~ 97.60), respectively. These data suggests that 15 μg or 7.5 μg dose of hemagglutinin antigen of the inactivated split-virion vaccine was safe and two doses of injection could induce a sufficient protective immune response in infants.

**Trial registration:**

Clinical trials registration: NCT01494740.

## Introduction

In April, 2009, a previously undescribed influenza A (H1N1) virus was isolated from people in Mexico and the United States, and then spreaded rapidly to many countries around the world. On June 11, 2009, a new influenza pandemic was officially declared by World Health Organization (WHO) (Dawood et al. 2009; World Health Organization 2009a). As of 18 July 2010, more than 214 countries around the world and overseas territories or communities have reported laboratory confirmed cases of pandemic influenza H1N1 2009, including over 18366 deaths (World Health Organization 2010a). During the outbreak, the children are mostly likely to be among the infected. Inconsistent with the traditional seasonal influenza, the 2009 influenza A (H1N1) virus is more likely to infect young people, rather than older people (Dawood et al. 2009; Echevarría-Zuno et al. 2009; Fraser et al. 2009; Domínguez-Cherit et al. 2009; Kumar et al. 2009; Donaldson et al. 2009). Across the reports of the 2009 influenza A (H1N1) virus infection cases, about 60% were young people less than 18 years of age, and the susceptibility of young children, which was further evidenced by the high hospitalization rates of those younger than 4 years (Centers for Disease Control and Prevention (CDC) 2009; Dawood et al. 2009).

The effectiveness of influenza vaccination in children to reduce the virus infection in the pandemic has been well demonstrated (Neuzil KM et al. 2001; Ruben 2004). Many countries and vaccine manufacturers have started to develop influenza vaccines against the 2009 influenza A (H1N1) virus, and evaluated the effect of vaccines in clinical trials. In Australia, the clinical trials have proved that one dose of the monovalent, split-virion vaccine without adjuvant was safe for people aged 18–64 years, and the seroconversion rates were 74.20% with 15 μg of hemagglutinin antigen dosage (Greenberg et al. 2009). In determining the 2009 influenza A (H1N1) vaccine was safe and could induce strong immune responses in healthy adults, the immune effect of vaccine in people of a small age group was studied further. In China, clinical trials have evaluated the safety and immunogenicity of the 2009 influenza A (H1N1) vaccine in people aged 3–60 years and our results have been published on the WHO website (Liang XF et al. 2010; Ze & Yang 2010). In addition, we also reported the long-term immunogenicity of the 2009 influenza A (H1N1) vaccine in the people aged 18–60 years (Yang et al. 2012). Subsequently, some papers reported the immune response in people under the age of 3 with 15 μg and 30 μg of hemagglutinin antigen dosage, which proved that the 2009 influenza A (H1N1) vaccine was safe and could induce enough immune response.

Our main purpose is to assess the safety and immunogenicity of the monovalent, unadjuvanted, split-virion 2009 influenza A (H1N1) vaccine in infants. In our study, infants under the age of 3 were divided into three age groups: 6–12 months, 13–24 months and 25–35 months in order to study the immune effects of the vaccine in infants. We described the immune responses of infants aged 6–35 months after being given 2 doses of injection with 7.5 μg and 15 μg of hemagglutinin antigen.

## Methods

### Study design

From December 2009 to January 2010, we carried out the double-blind, single-center clinical trial in Luxi County, Xiangxi Autonomous Prefecture, Hunan Province (China). The purpose of this study was to evaluate the safety and immunogenicity of the split-virion 2009 influenza A (H1N1) vaccine in healthy infants aged 6–35 months and these infants were divided into three groups: aged 6–12 months, 13–24 months and 25–35 months. All subjects received two doses of 7.5 μg, 15 μg hemagglutinin antigen or seasonal influenza vaccine with intervals three weeks apart. All of the pilot programs, clinical manuals and other materials used in this study were consistent with the Declaration of Helsinki and the quality control requirements for clinical trials, and were approved by the Ethics Committee of Hunan Province.

### Vaccines

The monovalent, inactivated, split-virion 2009 influenza A (H1N1) vaccine was developed by the Shanghai Institute of Biological Products, and the seed virus was prepared from the reassortant vaccine virus A/California/7/2009 NYMC X-179A, as recommended by the WHO (World Health Organization 2009b; World Health Organization 2010b). The vaccine was prepared in embryonated chicken eggs according to the standard techniques used in the production of seasonal influenza vaccine. In brief, the virus was amplified in chicken embryos, then harvested and inactivated with formaldehyde. The viral cultures were then concentrated and purified for use as the final vaccine. The experimental vaccines were split-virus products containing 7.5 μg of hemagglutinin antigen per dose (0.25 ml) and 15 μg of hemagglutinin antigen per dose (0.5 ml). The virus strains of the seasonal vaccine for children (SI) (A/Brisbane/59/2007 H1N1-like; A/Brisbane/10/2007 H3N2-like; and B/Brisbane/60/2008-like strains) were chosen according to the WHO recommendations for use in the 2009–10 influenza season (northern hemisphere winter). Vaccines contained 7.5 μg of hemagglutinin per strain per dose.

The vaccine was approved for clinical use by the Chinese National Institute for the Control of Pharmaceutical and Biological Products (China) and in full compliance with the Pharmacopoeia standards.

### Participants

All subjects must be strictly in line with the standard of ages 6–35 months. Infants with confirmed or suspected 2009 influenza A (H1N1) infection or those who had received other influenza products within 6 months were excluded from the study and the eligible infants did not have history of allergy or contraindications to the vaccine. All the infants were full-term babies and weighed more than 2500 grams. All subjects participated voluntarily in the clinical trials and their written informed consent was obtained from the parent or guardian of the infants. The blind testing was designed by a third party at Central South University, who was not involved in other elements of the clinical trials.

Eligible infants were stratified in three groups by age: aged 6–12 months with 52 subjects, 13–24 months with 130 subjects and 25–35 months with 130 subjects. After stratification, infants were randomized to receive the 7.5 μg, 15 μg of hemagglutinin antigen or seasonal influenza vaccine.

Injections were given intramuscularly in the deltoid muscle and 21 days after vaccination, the subjects were inoculated with the same dose of vaccine. Because the injection volume differed in the study doses, we have taken measures to avoid affecting the results of clinical trial. Serum samples were collected before or on days 21, 42 after vaccination.

### Safety

Induced local and systemic adverse events were recorded by the infant’s parent or guardian using a 7-day diary card, and other adverse events were recorded using a 21-day diary card. All local adverse events were considered related to the H1N1 vaccine. The systemic adverse events include: fever, allergic reactions, headache, fatigue, vomiting, diarrhea, coughing, crying, breastfeeding or eating disorders. The local adverse events include: (injection point) pain, swelling, redness, ecchymosis, induration, itching. All adverse events tests and decisions were referred to the standards of the Division of Microbiology and Infectious Diseases, US National Institutes of Health (NIAID Division of Microbiology and Infectious Diseases 2007a; NIAID Division of Microbiology and Infectious Diseases 2007b).

### Immunogenicity

The antibody titers against the vaccine strain were determined by HI assays of the anti-homologous strain of X-179A, performed in accordance with established measures using turkey erythrocytes. In brief, sera were firstly treated with receptor destroy enzyme at 36°C for 16 hours. The titers of HI antibody that were below the detection limit (i.e., <1:10) were recorded at a value of 1:5, and titers above 1:10240 were recorded at a value of 1:10240. The seroconversion rate represented a post-vaccination titer ≥1:40 in subjects with a pre-vaccination titer of <1:10 or a ≥4-fold titer increase in subjects with a pre-vaccination titer of ≥1:10. All serum samples were assayed in a blinded manner, in duplicate, and were checked in parallel by the Chinese National Institute for the Control of Pharmaceutical and Biological Products.

### Statistical analysis

For immunogenicity assessments, the seroconversion rate represented either a post-vaccination titer ≥1:40 (in accordance with the requirements for seasonal influenza vaccines by the European Committee for Proprietary Medicinal Products) in subjects with a pre-vaccination titer of <1:10 or a ≥4-fold titer increase in subjects with a pre-vaccination titer of ≥1:10. A seroprotection rate >70% was considered to provide protection. In addition, the geometric mean increase (GMI) was the ratio of the titer after vaccination to the titer before vaccination. All the serum data analysed in this research was from the subjects who received blood collections four times (European Agency for the Evaluation of Medicinal Products (EMEA) 1997).

Hypothesis testing was conducted using two-sided tests, with an alpha value of 0.05 considered to indicate statistical significance. All statistical analyses were performed using the SPSS software package (version 11.5).

## Results

### Study participants

A total of 312 subjects between 6 and 35 months of age participated in the clinical trial. Among them, 52 subjects were 6–12 months of age, 130 subjects were 13–24 months of age and 130 subjects were 25–35 months of age. Firstly, the 312 subjects were vaccinated and 312 serum samples were collected initially. Secondly, 265 subjects were vaccinated and 265 serum samples were collected. 25 subjects were gradually withdrawn from the clinical trial and 22 subjects refused to provide the serum. After 2 doses of injection, 252 serum samples were collected and 13 subjects refused to provide the serum. The specific vaccination data including gender, months of age are shown in Table 1. Of the 312 enrolled subjects, 252 subjects (80.77%) completed the entire study, providing three serum samples.

**Table 1:** **Participant disposition**

Dosage	Age	Dose	Subjects	Sex			Age, Mean
				Male, No.	Female, No.	Ratio (Male : Female)	
15 μg	6 ~ 12 m	1	20	10	10	1.00:1.00	9.70
		2	16	9	7	1.29:1.00	9.63
	13 ~ 24 m	1	50	25	25	1.00:1.00	19.24
		2	39	20	19	1.05:1.00	19.44
	25 ~ 35 m	1	50	26	24	1.08:1.00	29.54
		2	42	22	20	1.10:1.00	29.45
	Total	1	120	61	59	1.03:1.00	21.94
		2	97	51	46	1.11:1.00	22.15
7.5 μg	6 ~ 12 m	1	20	10	10	1.00:1.00	8.30
		2	19	9	10	0.90:1.00	8.42
	13 ~ 24 m	1	50	25	25	1.00:1.00	18.68
		2	41	21	20	1.05:1.00	18.71
	25 ~ 35 m	1	50	25	25	1.00:1.00	29.56
		2	44	22	22	1.00:1.00	29.75
	Total	1	120	60	60	1.00:1.00	21.48
		2	104	52	52	1.00:1.00	21.50
SI	6 ~ 12 m	1	12	6	6	1.00:1.00	9.83
		2	10	5	5	1.00:1.00	9.90
	13 ~ 24 m	1	30	15	15	1.00:1.00	18.53
		2	28	14	14	1.00:1.00	18.57
	25 ~ 35 m	1	30	15	15	1.00:1.00	29.23
		2	26	14	12	1.17:1.00	29.50
	Total	1	72	36	36	1.00:1.00	21.54
		2	64	33	31	1.06:1.00	21.66

### Safety of the vaccine

**Figure 1:**
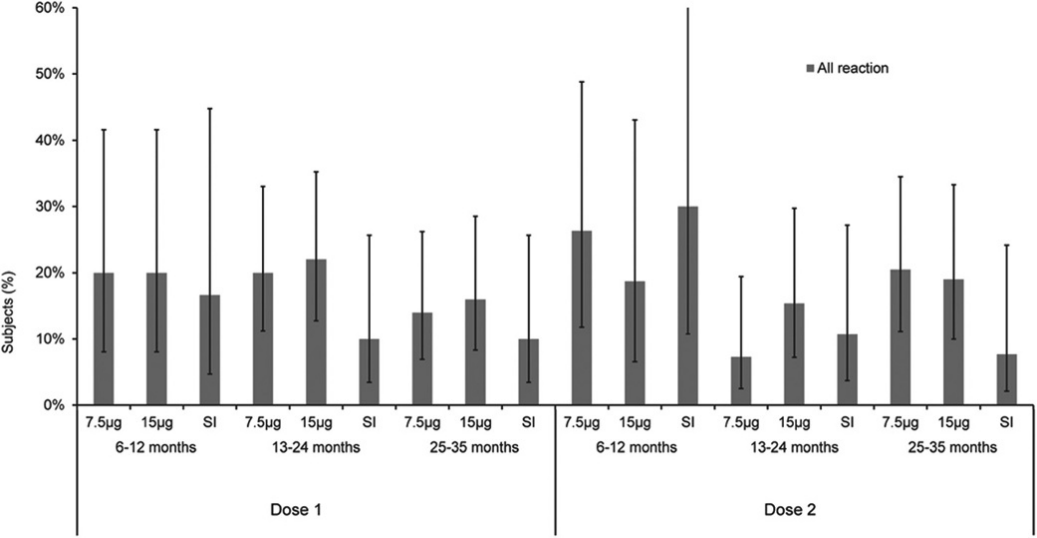
**Reactogenicity in all subjects: the total adverse reaction rates within 7 days after each vaccine dose.**

The overall adverse reaction rates are shown in Figure 1. After one dose of injection, the 7.5 μg group had a mild adverse reaction rate of 10.83% and the moderate adverse reaction rate of 6.67%. The 15 μg group had a mild adverse reaction rate of 10.83% and the moderate adverse reaction rate of 8.33%. The SI group had a mild adverse reaction rate of 6.25% and the moderate adverse reaction rate of 6.25%. In all groups, no serious adverse reactions were detected. Fever was the most frequently reported adverse effect. After one dose of injection, the fever reaction rate in the 7.5 μg, 15 μg and SI group were 11.67%, 13.33% and 9.72%, respectively. After two doses of injection, the fever reaction rate in the 7.5 μg, 15 μg and SI group were 14.42%, 15.46% and 12.50%, respectively. In addition to fever, eating disorders, vomiting, diarrhea, coughing, crying and other adverse reactions have occurred in the study dose group. Except for vomiting, other adverse reactions have occurred in the SI.

### Immune response

Before vaccination, the proportion of subjects showing HI ≥1:40 was 29.55% in the 6–12 months of age group with the highest observed, 18.27% in the 13–24 months of age group and 19.23% in the 25–35 months of age group (Table 2, Table 3).

**Table 2:** **Proportion of participants with seroprotection and seroconversion in the various groups**

Dosage	Age	Baseline	21 days after injection		42 days after injection	
		SP rate (95% CI)	SC rate (95% CI)	SP rate (95% CI)	SC rate (95% CI)	SP rate (95% CI)
15 μg	6-12 m	13.33	26.67	33.33	93.33	93.33
		(3.73 ~ 37.88)^b^	(10.90 ~ 51.95)	(15.17 ~ 58.28)	(70.18 ~ 98.81)^a^	(70.18 ~ 98.81)^a^
	13-24 m	32.43	51.35	67.57	94.59	94.59
		(19.63 ~ 48.53)^b^	(35.89 ~ 66.55)^a^	(51.47 ~ 80.37)^ab^	(82.29 ~ 98.50)^a^	(82.29 ~ 98.50)^a^
	25-35 m	21.05	55.26	65.79	89.47	94.74
		(11.07 ~ 36.34)	(39.70 ~ 69.85)^a^	(49.89 ~ 78.79)^a^	(75.86 ~ 95.83)^a^	(82.72 ~ 98.55)^a^
	Total	24.44	48.89	61.11	92.22	94.44
		(16.73 ~ 34.24)	(38.82 ~ 59.05)^a^	(50.78 ~ 70.53)^a^	(84.80 ~ 96.18)^a^	(87.64 ~ 97.60)^a^
7.5 μg	6-12 m	47.37	36.84	63.16	89.47	100.00
		(27.33 ~ 68.29)	(19.15 ~ 58.96)	(41.04 ~ 80.85)^a^	(68.60 ~ 97.06)^a^	(83.18 ~ 100.00)^a^
	13-24 m	9.76	34.15	39.02	80.49	82.93
		(3.86 ~ 22.55)^a^	(21.56 ~ 49.45)	(25.65 ~ 54.27)	(65.99 ~ 89.77)^a^	(68.74 ~ 91.48)^a^
	25-35 m	19.51	46.34	60.98	87.80	92.68
		(10.23 ~ 34.01)	(32.05 ~ 61.25)^a^	(45.73 ~ 74.35)^a^	(74.45 ~ 94.67)^a^	(80.57 ~ 97.48)^a^
	Total	20.79	39.60	52.48	85.15	90.10
		(14.02 ~ 29.70)	(30.61 ~ 49.35)^a^	(42.83 ~ 61.95)^a^	(76.93 ~ 90.79)^a^	(82.73 ~ 94.53)^a^
SI	6-12 m	20.00	0.00	20.00	10.00	30.00
		(5.67 ~ 50.98)	(0.00 ~ 27.75)	(5.67 ~ 50.98)	(1.79 ~ 40.41)	(10.78 ~ 60.32)
	13-24 m	11.54	3.85	15.38	3.85	15.38
		(4.00 ~ 28.98)	(0.68 ~ 18.90)	(6.15 ~ 33.53)	(0.68 ~ 18.90)	(6.15 ~ 33.53)
	25-35 m	16.00	0.00	16.00	12.00	28.00
		(6.40 ~ 34.65)	(0.00 ~ 13.32)	(6.40 ~ 34.65)	(4.17 ~ 29.96)	(14.28 ~ 47.58)
	Total	14.75	1.64	16.39	8.20	22.95
		(7.96 ~ 25.72)	(0.29 ~ 8.72)	(9.15 ~ 27.61)	(3.55 ~ 17.80)	(14.19 ~ 34.91)

^a^P < 0.05 compared with the SI group.

^b^P < 0.05 compared with the 7.5 dose group.

**Table 3:** **Geometric mean titer (GMT) and the geometric mean increase (GMI) in the various groups**

Dosage	Age	Baseline	21 days after injection		42 days after injection	
		GMT (95% CI)	GMT (95% CI)	GMI (95% CI)	GMT (95% CI)	GMI (95% CI)
15 μg	6-12 m	7.58	16.63	2.19	145.88	19.25
		(4.34 ~ 13.24)^b^	(8.11 ~ 34.07)^b^	(1.30 ~ 3.69)^b^	(80.39 ~ 264.73)^a^	(12.32 ~ 30.08)^a^
	13-24 m	13.75	63.90	4.65	255.56	18.59
		(9.03 ~ 20.94)	(37.46 ~ 108.97)^a^	(3.22 ~ 6.70)^a^	(170.61 ~ 382.91)^a^	(11.59 ~ 29.81)^a^
	25-35 m	10.37	65.45	6.31	226.26	21.82
		(6.50 ~ 16.55)	(40.06 ~ 106.95)^a^	(4.14 ~ 9.62)^a^	(151.81 ~ 337.21)^a^	(13.80 ~ 34.48)^a^
	Total	11.05	51.57	4.67	221.11	20.00
		(8.42 ~ 14.51)	(37.07 ~ 71.76)^a^	(3.63 ~ 6.00)^a^	(172.15 ~ 283.99)^a^	(15.19 ~ 26.34)^a^
7.5 μg	6-12 m	22.32	66.67	2.99	276.57	12.39
		(9.97 ~ 49.91)	(28.58 ~ 155.49)^a^	(1.85 ~ 4.81)^a^	(166.88 ~ 458.35)^a^	(7.16 ~ 21.44)^a^
	13-24 m	7.63	25.34	3.32	116.04	15.21
		(5.67 ~ 10.27)	(15.15 ~ 42.39)^a^	(2.24 ~ 4.93)^a^	(75.11 ~ 179.31)^a^	(10.57 ~ 21.89)^a^
	25-35 m	10.70	43.53	4.07	180.09	16.83
		(6.87 ~ 16.65)	(28.89 ~ 65.58)^a^	(2.85 ~ 5.80)^a^	(124.11 ~ 261.33)^a^	(11.20 ~ 25.30)^a^
	Total	10.71	37.86	3.54	163.34	15.25
		(8.22 ~ 13.95)	(27.83 ~ 51.50)^a^	(2.82 ~ 4.44)^a^	(127.06 ~ 209.94)^a^	(12.03 ~ 19.33)^a^
SI	6-12 m	9.33	8.71	0.93	14.14	1.52
		(3.62 ~ 24.08)	(3.77 ~ 20.09)	(0.80 ~ 1.09)	(4.70 ~ 42.60)	(0.69 ~ 3.31)
	13-24 m	7.87	8.52	1.08	12.05	1.53
		(4.68 ~ 13.22)	(5.51 ~ 13.18)	(0.83 ~ 1.41)	(7.67 ~ 18.94)	(1.10 ~ 2.13)
	25-35 m	8.71	8.01	0.92	14.74	1.69
		(5.30 ~ 14.29)	(5.19 ~ 12.37)	(0.81 ~ 1.04)	(8.57 ~ 25.35)	(1.19 ~ 2.41)
	Total	8.43	8.34	0.99	13.44	1.59
		(6.14 ~ 11.58)	(6.35 ~ 10.95)	(0.87 ~ 1.12)	(9.74 ~ 18.54)	(1.28 ~ 1.99)

^a^P < 0.05 compared with the SI group.

^b^P < 0.05 compared with the 7.5 dose group.

Immune responses were induced in all subjects after vaccination. After one dose of injection, the rates of seroconversion and seroprotection in the 7.5 μg group were 39.60% and 52.48%, and the HI Geometric mean titer (GMT) growth multiple was 37.86. The rates of seroconversion and seroprotection in the 15 μg group were 48.89% and 61.11%, and the HI GMT was 51.57. After two doses of injection, the rates of seroconversion and seroprotection in the vaccine groups were all increased. The rates of seroconversion and seroprotection in the 7.5 μg group were 85.15% and 90.10%, and the HI GMT was 163.34. The rates of seroconversion and seroprotection in the 15 μg group were 92.22% and 94.44%, and the HI GMT was 221.11.

After one dose of injection, in the 7.5 μg group, the rates of seroconversion and seroprotection were 36.84% and 63.16% in the 6–12 months of age group, 34.15% and 39.02% in the 13–24 months of age group, 46.34% and 60.98% in the 25–35 months of age group. Except the rate of seroprotection in the 13–24 months of age group was lower than that of the other two groups, the rates of seroconversion and seroprotection in the 6–12 months and 25–35 months of age groups all had no significant difference. And in the 15 μg group, the rates of seroconversion and seroprotection were 26.67% and 33.33% in the 6–12 months of age group, 51.35% and 67.57% in the 13–24 months of age group, 55.26% and 65.79% in the 25–35 months of age group. Except for the rate of seroprotection in the 6–12 months of age group that was lower than that of the other two groups, the rates of seroconversion and seroprotection in the 13–24 months and 25–35 months of age groups all had no significant difference. The difference in the rates of seroprotection in the different months of age groups may be related to the difference in the baseline antibody titers.

After two doses of injection, in the 7.5 μg group, the rates of seroconversion and seroprotection were 89.47% and 100.00% in the 6–12 months of age group, 80.49% and 89.93% in the 13–24 months of age group, 87.80% and 92.68% in the 25–35 months of age group. The rates of seroconversion and seroprotection in the three months of age groups all had no significant difference. And in the 15 μg group, the rates of seroconversion and seroprotection were 93.33% and 93.33% in the 6–12 months of age group, 94.59% and 94.59% in the 13–24 months of age group, 89.47% and 94.74% in the 25–35 months of age group. Similarly, the rates of seroconversion and seroprotection in the three months of age groups all had no significant difference.

In the SI group, the proportion of HI ≥1:40 post-vaccination had increased slightly compared to the values of pre-vaccination (14.75%). The protection rate was 16.39% after one dose of injection and 22.95% after two doses of injection. Similarly, the GMT of the HI antibody titers was 8.34 after one dose of injection and 13.44 after two doses of injection.

In summary, in the subjects administered with two doses of 7.5 μg vaccine, the rates of seroconversion and seroprotection met the requirements specified by the EMEA (The European Agency for the Evaluation of Medicinal Products). The results showed that the vaccine could provide protection against the 2009 H1N1 virus.

## Discussion

It is well-known that influenza can cause infection in different age stratification and spread fast. However, children and the elderly are more susceptible to influenza virus and the rates of infection are highest among children resulting in serious illness and death. In infants below 2 years old, the hospitalization rates are much higher than the older infants and children, and the risk of receiving influenza-related complications are also higher than that of other age groups (Fiore et al. 2008). So, the infants are the primary targets of the influenza vaccine to control influenza pandemic.

Some papers have reported the safety and immunogenicity of the 2009 influenza A (H1N1) vaccine in people aged >3 years old. The results showed that a single 7.5 μg of this monovalent 2009 influenza A (H1N1) vaccine can induce enough antibody responses (Greenberg et al. 2009; Liang XF et al. 2010). However, Walter EB indicated that, due to the immature immune system or not infected by the influenza virus, the vaccine induced significant lower antibody responses in infants aged <1 year old (Walter EB et al. 2010). Thus, it is necessary to study the immunogenicity of the 2009 influenza A (H1N1) vaccine in infants. Previous studies have explored the immune effect of vaccine in infants aged <3 years old. In the UK, after two 7.5 μg doses of injection with the whole virus influenza vaccine in infants aged <12 years old, the proportion of subjects showing HI ≥1:32 was 78.20% (Waddington CS et al. 2010). Among them, the proportion of subjects showing HI ≥1:32 was 65.70% in 6 months to 3 years of age. In Australia, a single 15 μg of the inactivated split-virion H1N1 vaccine caused a significant increase of the specific antibody in 86.78% of the infants aged 6 months to 9 years (Nolan et al. 2010). In South Korea, after one 7.5 μg dose of injection with the inactivated split-virion H1N1 vaccine in infants aged 6 months to 3 years, the proportion of subjects showing HI ≥1:40 was 55.90% (Chi-Eun et al. 2010).

Our study demonstrated that a second 7.5 μg dose of this monovalent 2009 influenza A (H1N1) vaccine can induce stronger antibody responses in infants (aged 6 months to 3 years). After immunization, the rates of seroprotection and seroconversion were 90.10% and 85.15% respectively and the GMI was 15.25. The above results met the standard of vaccine immunogenicity required by European Union Committee for Medicinal Products.

Similar to many results reported previously, a higher than expected proportion of infants exhibited baseline antibody titers greater than 1:40 (Greenberg et al. 2009; Nolan et al. 2010). While we excluded infants with confirmed H1N1 infection, these results illustrated the high number of recessive infection in infants. Our study was carried out in 8 months after the outbreak of the H1N1 virus, which increased the possibility of the people subjected with recessive infected with influenza virus. In addition, the results showed that the proportion of the people recessive infected with influenza virus was different in different areas of China.

In the SI group, the proportion of HI antibody against 2009 H1N1 virus ≥ 40 was 16.39% after one dose of injection and 22.95% after two doses of injection. The seasonal vaccine did not provide protection against 2009 H1N1 virus in infants. These results were consistent with those reported in other papers that little cross-reactive antibody against the 2009 H1N1 virus after seasonal vaccination (Centers for Disease Control and Prevention (CDC) 2009).

In our study, the proportion of infants aged 6 months to 3 years showing baseline antibody titers greater than 1:40 was 20.63%. After one dose of 7.5 μg vaccine, the rates of antibody titers greater than 1:40 in three age groups were 63.16%, 39.02% and 60.98%, respectively. After one dose of 15 μg vaccine, the rates of antibody titers greater than 1:40 in the three age groups were 33.33%, 67.57% and 65.79%, respectively. These results were higher than those reported by the South Korea and lower than those reported by the Australia, which may be influenced by the baseline antibody titers (Chi-Eun et al. 2010; Nolan et al. 2010). The baseline antibody titer of different months of age groups had significant differences. After one dose of injection, the rates of seroprotection in the three months of age groups had significant differences. After two doses of injection, the rates of seroconversion and seroprotection in the three months of age groups had no significant difference. The above results showed that only two doses of injection of the monovalent 2009 influenza A (H1N1) vaccine could induce enough antibody responses in infants and also avoided the difference of the rates of seroprotection caused by the baseline antibody titer. In addition, the results also verified the recommendation of the Centers for Disease Control and Prevention, which the Advisory Committee on Immunization Practices suggested that infants and children aged 6 months to 9 years should receive 2 doses of H1N1 influenza vaccine.

Our study showed that 7.5 μg dose of the monovalent 2009 influenza A (H1N1) vaccine was well tolerated and immunogenic, which could provide protection for infants and reduce the spread of the virus in the influenza pandemic.

## Abbreviations

CI: Confidence interval
EMEA: European agency for the evaluation of medicinal products
GMI: Geometric mean increase
GMT: Geometric mean titer
HI: Hemagglutinin inhibition
SC: Seroconversion
SI: Seasonal influenza vaccine
SP: Seroprotection
WHO: World health organization.
